# The Canadian Occupational Performance Measure: a Feasible Multidisciplinary Outcome Measure for Pediatric Telerehabilitation

**DOI:** 10.5195/ijt.2021.6372

**Published:** 2021-06-22

**Authors:** Lynn R. Tanner, Kathy Grinde, Cristin McCormick

**Affiliations:** 1 Physical Medicine & Rehabilitation, Children's Minnesota, Minneapolis, Minnesota, USA

**Keywords:** Child, Occupational therapy, Physical Therapy, Speech-Language Pathology, Telerehabilitation

## Abstract

This study describes the feasibility of using the Canadian Occupational Performance Measure (COPM) as a multidisciplinary outcome measure for pediatric telerehabilitation (TR). The COPM was administered at monthly time points over four months. A follow-up survey was conducted with the therapists to assess clinical utility of the COPM. Seventy-three percent of the children seen in TR > one month had at least two administrations of the COPM. Eighty percent of therapists agreed or strongly agreed that the COPM was easy to use in a reasonable amount of time, helped identify functional goals, could be used with various children with varied diagnoses, and measured functional change. In 37 children, the median clinical change in performance and satisfaction was two points or greater on the COPM over the episode of TR. The COPM is a feasible measure perceived positively by pediatric therapists for TR use.

Children across the world receive pediatric rehabilitation services secondary to functional impairments impacting quality of life. The Coronavirus (COVID-19) pandemic brought imminent challenges to the rehabilitation community providing occupational therapy (OT), physical therapy (PT), and speech-language pathology (SLP) services to children, adolescents, and young adults. To comply with stay-at-home orders to reduce the spread of the disease and to reduce use of personal protective equipment, many institutions responded with the long-awaited offering of telerehabilitation (TR) to provide these services safely and responsibly. While many terms are used in the field of telehealth, the American Telemedicine Association defines telerehabilitation as “the delivery of rehabilitation and habilitation services via a variety of communication technologies….that include evaluation, assessment, monitoring, prevention, intervention, supervision, education, consultation and coaching” (Richmond et al., 2017). In the pediatric realm, therapists provide these services through the educational system or health care systems.

While the pandemic accelerated the adoption of TR secondary to necessity and increased reimbursement for the services, researchers have investigated the benefits over the last decade at a minimum. A systematic review completed in 2008 included 28 articles primarily encompassing adult rehabilitation and found positive clinical outcomes utilizing TR interventions that were similar or better than comparison interventions (Kairy, et al., 2009). More recently, Camden et al. (2020) completed a systematic review in children with disabilities, finding 23 articles with many interventions and populations. Interestingly, over half of the outcome measures improved with TR, often through coaching interventions aimed at caregiversand utilizing exercise programs (Camden et al., 2020). A variety of health care professionals and a wide range of outcome measures were represented in the data.

Very few outcome measures are valid for use by multiple disciplines. The Canadian Occupational Performance Measure (COPM) is accepted for use by occupational therapists and allied health professionals (Law et al., 2005). It has been identified as an effective multidisciplinary tool for measuring outcomes in the early childhood population and has been administered by both occupational and physical therapists in a broad range of pediatric research studies (Calder et al., 2018; Carswell et al., 2004; Heus et al., 2020; Mirek et al., 2019).

Speech and language therapists have used the tool as well (Raghavendra, et al., 2015). The COPM was found to be appropriate for TR with a small group of adults, as it relies on verbal responses, not physical movement (Dreyer, et al. 2001). It has also been used as an outcome measure in previous pediatric telehealth research, though on a limited basis (Camden et al., 2020; Hung Kn & Fong, 2019; Steinhart, et al., 2020).

As pediatric TR grows, there is a need to identify appropriate outcome measures that can be used to assess the quality of care that is being provided by multiple disciplines. Pediatric rehabilitative care is grounded in the model of family-centered care (Coyne, et al., 2018). The COPM is an outcome measure that integrates child and family perceptions and goals into the care plan. The purpose of this quality improvement initiative was to understand and describe the feasibility of using the COPM as a multidisciplinary outcome measure for pediatric TR. Is it feasible for PT, OT, and SLP to use the COPM as a patient-reported outcome measure in children receiving pediatric TR? The secondary aim was to describe the initial trends of change in COPM scores during an episode of TR care.

## METHODS

**Figure 1:**
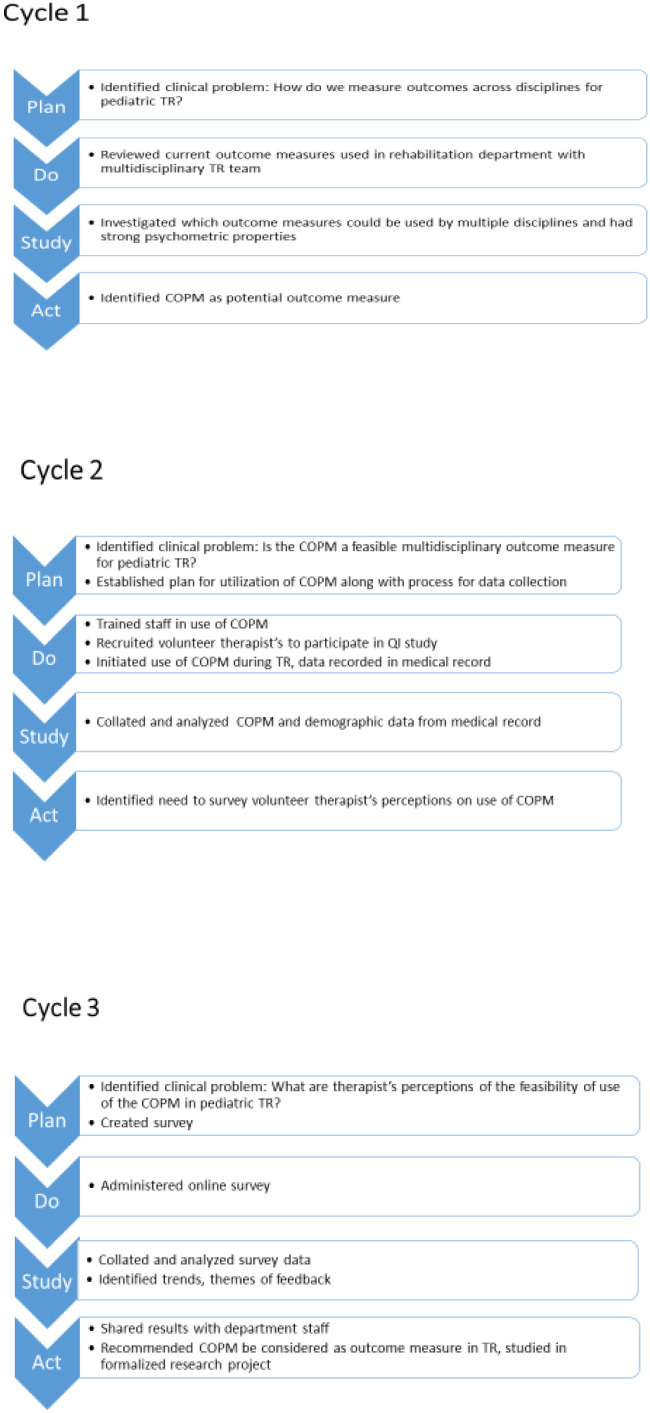
QI Study Process

Our study took place in a pediatric outpatient hospital-based system during the spring and summer of 2020 during the COVID-19 pandemic. The process for the quality improvement (QI) project followed “plan-do-study-act” cycles (Wong & Sullivan, 2016) (Figure 1) and SQUIRE guidelines were used for reporting (Ogrinc et al., 2016). All therapists were trained in the use and delivery of TR services via videoconferencing software for pediatric rehabilitation populations before the study. TR treatment incorporated family-centered care principles, which included individualized goal-oriented care plans created in collaboration with the child and family with consideration given to their family's background and culture (Coyne et al., 2018). The need for a measurement tool that would help assess the quality of TR treatment with feedback from the child and caregivers was identified.

The COPM was chosen as it has established psychometric properties for validity, test-retest reliability, and responsiveness to change in pediatric populations (Carswell et al., 2004; Cusick, et al., 2007; Sakzewski, et al., 2007). It was also available to staff at all sites and supported the core values of family-centered care. The COPM is an individualized tool that helps set treatment goals by identifying challenges that a child encounters in daily activities (Law et al., 2005). Baseline scores with a range of one to ten are scored to assess participation and satisfaction on identified goals. These scores are compared to subsequent scores to determine perceived outcomes. A change of two or more points is deemed clinically meaningful (Law et al., 2005). The COPM can be used with a wide variety of patient diagnoses and ages (Law et al., 2005). The test is valid to administer with a parent or guardian when a child is too young (Law et al., 2005) or incapable of identifying goals (Verkerk, et al., 2006). The measure requires verbal feedback alone and no physical or observed measurement.

**Figure 2:**
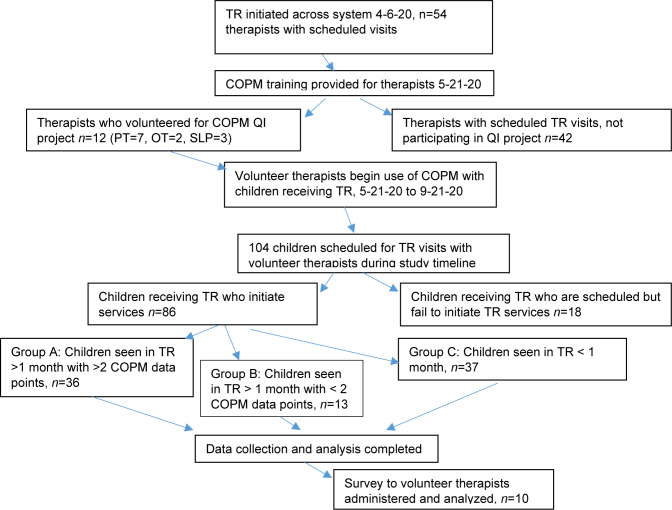
Flow Diagram COPM QI study

Therapists from multiple disciplines (OT, PT, SLP) were provided education on using the COPM as an outcome measure and invited to volunteer for the project. Twelve out of a total of 54 therapists providing TR volunteered to participate in the QI project with representation from all three disciplines (Figure 2). Their practice experience ranged from 1 year to 32 years. All volunteers completed the COPM training provided by the department. Two of the therapists had used the COPM extensively, with four using it “a little” and four with no prior experience. The therapists were asked to complete the COPM with the children and/or caregivers they were working with via TR and record the scores every month over a period of four months. Therapists recorded data in the medical record. The QI lead emailed monthly reminders to collect and record data to the participating therapists to enhance compliance. Demographics of the children who received TR treatment are noted in Table 1.

**Table 1:** Participant Demographics

Characteristic	Group A	Group B	Group C
	Children seen in TR >1 month with >2 COPM data points, n=36	Children seen in TR > 1 month with < 2 COPM data points, n=13	Children seen in TR < 1 month, n=37
Age (years)			
Mean (STD)	8.13(6.71)	7.21(5.31)	6.24(4.95)
Median	6.04	6.46	4.67
Age (years)			
<2	6 (17%)	2 (15%)	6 (16%)
2-6	15 (42%)	4 (31%)	20 (54%)
7-12	5 (14%)	5 (38%)	6 (16%)
13-22	10 (28%)	2 (15%)	5 (14%)
Diagnosis			
Neurodevelopmental	16 (44%)	9 (69%)	22 (59%)
Oncology	11 (31%)	3(23%)	9 (24%)
Torticollis	5 (14%)	0 (0%)	4 (11%)
Chronic pain	2 (6%)	0 (0%)	0 (0%)
Concussion	1 (3%)	0 (0%)	0 (0%)
Cardiac	1 (3%)	1 (8%)	2 (5%)
Sex			
Male	21 (58%)	7 (54%)	24 (65%)
Female	15 (42%)	6 (46%)	13 (35%)

The investigators designed a survey with a focus on demographics of the therapists, perceived ease of use, applicability to the populations served, and relationship to functional change. It was administered to the participating therapists within one week following completion of the study. It used a five-point Likert scale with defined criteria for each point on the scale.

## DATA ANALYSIS

Data were gathered from the medical record, de-identified, and analyzed at the end of the 4-month time frame. The feasibility of implementation was assessed by analyzing the frequency and consistency of use of the COPM over time with children scheduled for TR with the volunteer therapists. The clinical utility of the COPM for TR was assessed with content analysis of the therapist survey. Trends in the data were studied in a secondary analysis, which included the children's demographics, standard deviation, and median change on the COPM for all children with two or more data points.

The study's scope and aims were evaluated by the institutional review board at our institution and determined to qualify for an exemption.

## RESULTS

## FEASIBILITY

Twenty-two percent of the therapists performing TR at the time of the study volunteered to participate (Figure 2). Eighty-two percent of the children scheduled for TR services attended a TR visit. Therapists saw forty-three percent of the remaining children for less than one month of treatment, which did not allow for collecting two COPM data points (Group C). The COPM was completed two or more times with seventy-three percent of the children seen in TR for greater than one month (Group A), though only eight of these patients had more than two COPM data points. Twenty-seven percent of the children were seen for more than one month but did not have more than two COPM data points (Group B).

**Table 2:** Descriptive Measures

Measure	Group A (n=36)	Group B (n=13)	Group C (n=37)
	Children seen in TR >1 month with >2 COPM data points	Children seen in TR > 1 month with < 2 COPM data points	Children seen in TR < 1 month
COPM			
Performance			
Mean (STD)	2.34 (1.76)	NA	NA
Median	2		
Satisfaction			
Mean (STD)	2.30 (2.04)	NA	NA
Median	2		
Number of TR visits during study period			
Mean (STD)	5.86 (3.82)	6.08(2.64)	1.67(1.13)
Median	5	6	1
Number of days in TR treatment			
Mean (STD)	56.89 (29.94)	66.38(24.24)	1(6.25)
Median	50	61	1

## CLINICAL UTILITY

**Figure 3:**
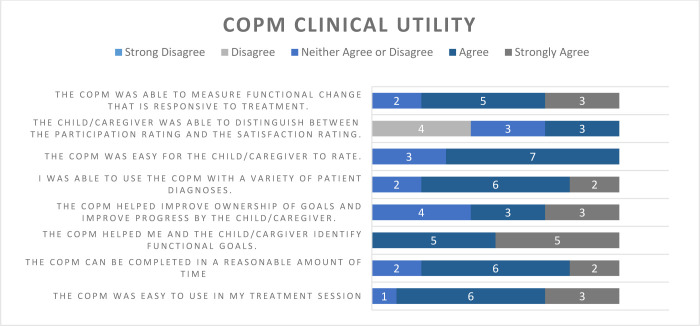
COPM Survey Responses

Ten of the twelve therapists (83%) in the project responded to the survey, with representation from all three disciplines. Eighty percent or more of the respondents agreed or strongly agreed that the COPM was easy to use in a reasonable amount of time, helped identify functional goals, could be used with a variety of children with varied diagnoses, and was able to measure functional change (Figure 3). Reasons given for not using the COPM for children in Group B included: the therapist was changing roles in the department, or the child was expected to switch to in-person visits.

## TRENDS IN DEMOGRAPHICS AND COPM DATA

Children were seen from a wide range of ages (Table 1) and multiple diagnostic categories in Groups A and B. Group A had a slightly higher percentage of children in the 2-to-6-year age group and 13-to 22-year age group. There were slightly more males than females in both groups. Group A had a wider range of diagnostic categories than Group B though oncology and neurodevelopmental diagnosis were the top categories in both groups. Clinically meaningful change (>two points) was reported for 56% of patients regarding Performance and 52% for Satisfaction in Group A. The mean and median change for Group A children was two points or greater on both measures of Participation and Satisfaction.

## DISCUSSION

Due to the rapid growth of telemedicine, and in this case, TR via videoconferencing, it is imperative to quantify the quality of rehabilitation services. Having a patient-reported outcome that can be utilized across disciplines in a time-efficient and purposeful manner without physical measurement allows for efficient analysis of service quality. This quality improvement initiative demonstrated adequate feasibility of the use of the COPM across disciplines in pediatric TR. Two observations in this initiative point towards the COPM as a promising outcome in TR: (1) the positive response of therapists regarding its utility across a multidisciplinary team, and (2) the ability to complete the assessment at least two times in over 70% of the appropriate patients during the initial 6 months of a TR program. The initial findings support exploring the use of the COPM on a broader scale in pediatric TR.

Therapists from all three rehabilitation disciplines reported the positive clinical utility of the COPM in this small sample. This is consistent with the recommendation of the COPM for use in multidisciplinary pediatric practice, such as early intervention services (Calder, et al., 2018) and children with cerebral palsy of school age (Sakzewski et al., 2007). Importantly, with the rapid uptake of TR during the COVID-19 pandemic and the need for effective outcome measures to use across a wide variety of pediatric patient populations, the therapists found the COPM effective and easy to use across different ages and diagnoses. While clinical effectiveness of the COPM cannot be investigated in this feasibility QI initiative, the positive trend observed in the change in participation and satisfaction with the median and mean score over the clinically meaningful difference is promising. Further research using the COPM in both adult and pediatric TR compared to clinical measures (in-person and using TR) is necessary to investigate utilizing the COPM to determine TR's efficacy or effectiveness.

The therapist survey results demonstrated concern regarding the caregiver or child's ability to distinguish between the participation and the satisfaction rating for goals. Other research has also highlighted this concern when using the COPM (Eyssen et al., 2011). A possible solution to assist in understanding may be to explain the COPM through the patient portal before its use in this setting, or screen sharing an explanation of the two ratings during testing. The amount of time between COPM administrations during the data collection suggests that the frequency of one time per month was possibly too high. Therapists commented that this frequency was appropriate for children with a diagnosis such as torticollis or concussion, but not appropriate for children with neurodevelopmental diagnoses secondary to a slower rate of expected progress. Clinically, the COPM is typically given at initiation of services, reassessment periods, near discharge, or when new goals are developed. The next improvement cycle could recommend the frequency of COPM administration based on prognostic rate of change.

## STUDY LIMITATIONS

There were multiple limitations to this QI initiative when considering the next steps of clinical improvement or generalizability into a multidisciplinary context. This QI initiative did not address parent/caregiver satisfaction of the use of the COPM; however, previous literature in small samples demonstrates that caregivers value goal setting within the realm of pediatric rehabilitation (Øien, et al, 2010) and also value the use of the COPM to measure the impact of an intervention (Verkerk, et al., 2020). Future research should explore whether goal setting with the COPM translates to families in the same manner in the TR mode of care as in-person rehabilitation. Secondary to a period of high stress within a pandemic, the number of volunteers for the QI initiative was low, with a higher proportion of PTs. A substantial group of children received less than 1 month of TR following staffing changes. Also, a 4-month time frame was chosen for data collection due to the swift introduction of a new mode of rehabilitation; however, a longer time span would have allowed for outcomes on more children to better explore TR's impact using the COPM. Completing this QI project again once the health system is stabilized outside of a global pandemic is recommended as health care delivery may have differed during a pandemic.

## CONCLUSION

With the increase in TR services provided during the COVID-19 pandemic and reported caregiver satisfaction of pediatric TR (Tenforde et al., 2020), it is essential to establish the reliability and validity of outcome measures in the pediatric population to assure high-quality care that is comparable or better than in-person care. The COPM has been used in pediatric TR in the research setting successfully (Ferre et al., 2017); however, the validity of the measure in this mode of care has not been investigated to the authors' knowledge. Multiple tools have been deemed reliable and valid in TR physical therapy for musculoskeletal disorders (Mani, et al., 2017); however, there is less research available for TR in the pediatric population. Some outcome measures are validated in pediatric TR such as the Clinical Evaluation of Language Fundamentals (4^th^ edition) and the Speech Intelligibility Rating for Pediatric Language Assessment (Taylor et al., 2014) or the Movement Assessment Battery for Children (2^nd^ edition) for motor performance (Nicola, et al., 2018); however, these tests are not able to detect change across the entire rehabilitation discipline such as the COPM. While there is much to be learned regarding pediatric TR, this quality improvement initiative supported further use and study of the COPM as a family-centered outcome measure.
